# Characteristics of trunk and lower limb alignment at maximum reach during the Star Excursion Balance Test in subjects with increased knee valgus during jump landing

**DOI:** 10.1371/journal.pone.0211242

**Published:** 2019-01-25

**Authors:** Kazuma Uebayashi, Kiyokazu Akasaka, Akihiro Tamura, Takahiro Otsudo, Yutaka Sawada, Yu Okubo, Toby Hall

**Affiliations:** 1 Department of Rehabilitation, Suzuki Clinic Orthopaedics River City, Chuo, Tokyo, Japan; 2 Saitama Medical University Graduate School of Medicine, Moroyama, Saitama, Japan; 3 School of Physical Therapy, Saitama Medical University, Moroyama, Saitama, Japan; 4 Department of Physical Therapy, School of Health Sciences at Narita, International University of Health and Welfare, Narita, Chiba, Japan; 5 School of Physiotherapy and Exercise Science, Curtin University, Perth Western Australia, Bentley, Australia; Mayo Clinic Rochester, UNITED STATES

## Abstract

**Background:**

The anterior cruciate ligament (ACL) is often injured during sport. The Star Excursion Balance Test (SEBT) has been used to evaluate ankle and knee stability of the supporting leg while reaching in eight different directions with the non-stance leg. We hypothesized that the SEBT might be useful in categorising ACL injury risk. The purpose of this study was to clarify the relationship between knee valgus alignment during single leg drop landing (SDL) and alignment of the trunk and lower limb during the SEBT.

**Methods:**

A three-dimensional motion analysis system was used to measure the trunk, hip and knee angles during SDL and the SEBT. Groupings were allocated based on 5 degrees of knee valgus angle during SDL. Independent t-test’s were used to identify differences in the trunk, hip and knee angles between the two groups.

**Results:**

The knee valgus angles in the knee valgus group were greater than those in the control group in five directions of the SEBT (p < 0.05). In addition, the hip internal rotation angle in the knee valgus group was lower than that in the control group during two directions of the SEBT (p < 0.05). Furthermore, the knee flexion and trunk right rotation angles in the knee valgus group were lower than those in the control group in two directions of the SEBT (p < 0.05).

**Conclusion:**

Decreases in hip internal rotation, knee flexion and trunk rotation to the supporting leg during the SEBT might be considered as risk factors for non-contact ACL injury.

## Introduction

Anterior cruciate ligament (ACL) injury is a serious and potentially devastating injury for athletes, and is common in many sports including soccer, basketball, handball and lacrosse. Injuries can be classified as either contact or non-contact with up to 70% classified as non-contact [1–5]. While non-contact ACL injury prevention programs have been widely studied, Distefano et al. suggested that ACL injury prevention programmes should be matched to the ACL injury risk of participants and designed along these characteristics [6]. Therefore, it is important to establish an evaluation method where the risk of non-contact ACL injury can be classified.

One of the most significant mechanism’s for non-contact ACL injury risk has been widely considered poor alignment of the trunk and lower limbs [7 – 9]. Hewett et al., in their prospective study, reported that knee valgus when landing from a drop vertical jump increased the risk of non-contact ACL injury [10]. In addition, prospective studies have reported that increased knee valgus angle and knee abduction moment during landing were predictive of non-contact ACL injuries in female athletes [3, 10, 11]. Furthermore, knee valgus has been reported as a component of mal-alignment in non-contact ACL injury situations. Krosshuag et al. reported that greater knee valgus and less hip and knee flexion made athletes vulnerable to injury during landing and cutting [12]. Hewett et al. reported that greater knee valgus and greater trunk flexion to the supporting leg were factors placing athletes at high risk of non-contact ACL injury also during landing and cutting [13], while Koga et al. reported that less knee flexion and greater knee valgus during landing and cutting placed athletes at a high risk of injury [14]. Although dynamic knee valgus during landing is considered to be important for prediction of non-contact ACL injuries, various characteristics of trunk and lower limb alignment have also been confirmed to influence knee valgus at the time of injury. Therefore, an evaluation of these features including knee valgus might be helpful in planning injury prevention programs.

Stability and distance reached during the Star Excursion Balance Test (SEBT) has been commonly used as an assessment tool to determine postural control and lower extremity injury risk [15–18]. In this test, the non-stance leg reaches as far as possible along eight lines drawn on the ground at 45 degrees to each other. Although there is some research reporting the relationship between the reach distance during the SEBT and injury risk after ACL reconstruction surgery [19,20], alignment of the lower limb related to non-contact ACL injury risk during this demanding task has not been investigated. We investigated whether altered alignment of the trunk and supporting leg during the SEBT and increased knee valgus alignment during single leg drop landing (SDL) might be associated and if so the SEBT might be helpful in identifying people vulnerable to non-contact ACL injury. Therefore, the purpose of this study was to investigate the difference in trunk and supporting leg alignment during the SEBT in people with and without dynamic knee valgus during a jump landing task.

## Materials and methods

### Participants

Twenty-eight healthy females volunteered to participate in this study (Table 1). Participants were recruited with no history of orthopaedic spine, hip, knee and ankle surgery as inclusion criteria. The dominant foot was the right in 27 participants and the left in 1 participant. The dominant foot was determined based on which foot was used to kick a ball [21]. All participants gave written informed consent prior to the start of the study. The study protocol followed the Helsinki declaration and was approved by the Ethics Committee at the Saitama Medical University, Saitama, Japan (M– 62). The individual in this manuscript has given written informed consent (as outlined in PLOS consent form) to publish these case details.

**Table 1:** The results of comparisons of participants’ characteristics, namely knee valgus or varus angle, age, height and weight, between valgus and control groups.

	Valgus group (n = 10)	Control group (n = 18)	p value
Knee valgus or varus angle during SDL (mean± SD, deg) ^a^	−8.2 ± 2.9	4.6 ± 6.0	<0.01^b^
Age (mean± SD, years)	20.4 ± 1.1	20.7 ± 1.3	0.59
Height (mean± SD, cm)	157.8 ± 4.0	160.2 ± 5.0	0.21
Weight (mean± SD, kg)	50.7 ± 2.9	51.6 ± 5.0	0.63

^a^ Positive value indicates the knee varus angle, and negative value indicates the knee valgus angle.

^b^ Statistically significant at p < 0.05.

### Measurement

A three-dimensional motion analysis system with eight cameras (Vicon MX; Vicon Motion Systems) was used to measure trunk and lower extremity angle data during single-leg drop landing (SDL) and the SEBT. The sampling rate for angle data was set at 240 Hz. Thirty-five reflective markers were placed on specific anatomical landmarks (left and right front and back heads, 7th cervical vertebrae, 10th thoracic vertebrae, clavicle, sternum, right back, shoulders, lateral epicondyles of the elbow, medial wrists, lateral wrists, second metacarpal heads, anterior superior iliac spines, posterior superior iliac spines, lateral thighs, lateral epicondyles of the knee, lateral thigh tibias, lateral malleoli, second metatarsal heads and heels) to measure trunk and lower extremity angle data using Plug in Gait Marker Full Body Model. Two force plates (AMTI MSA-6) were used to record the ground reaction force (GRF) during SDL. The sampling rate for GRF data was set at 1200 Hz.

### Single leg drop landing

**Fig 1:**
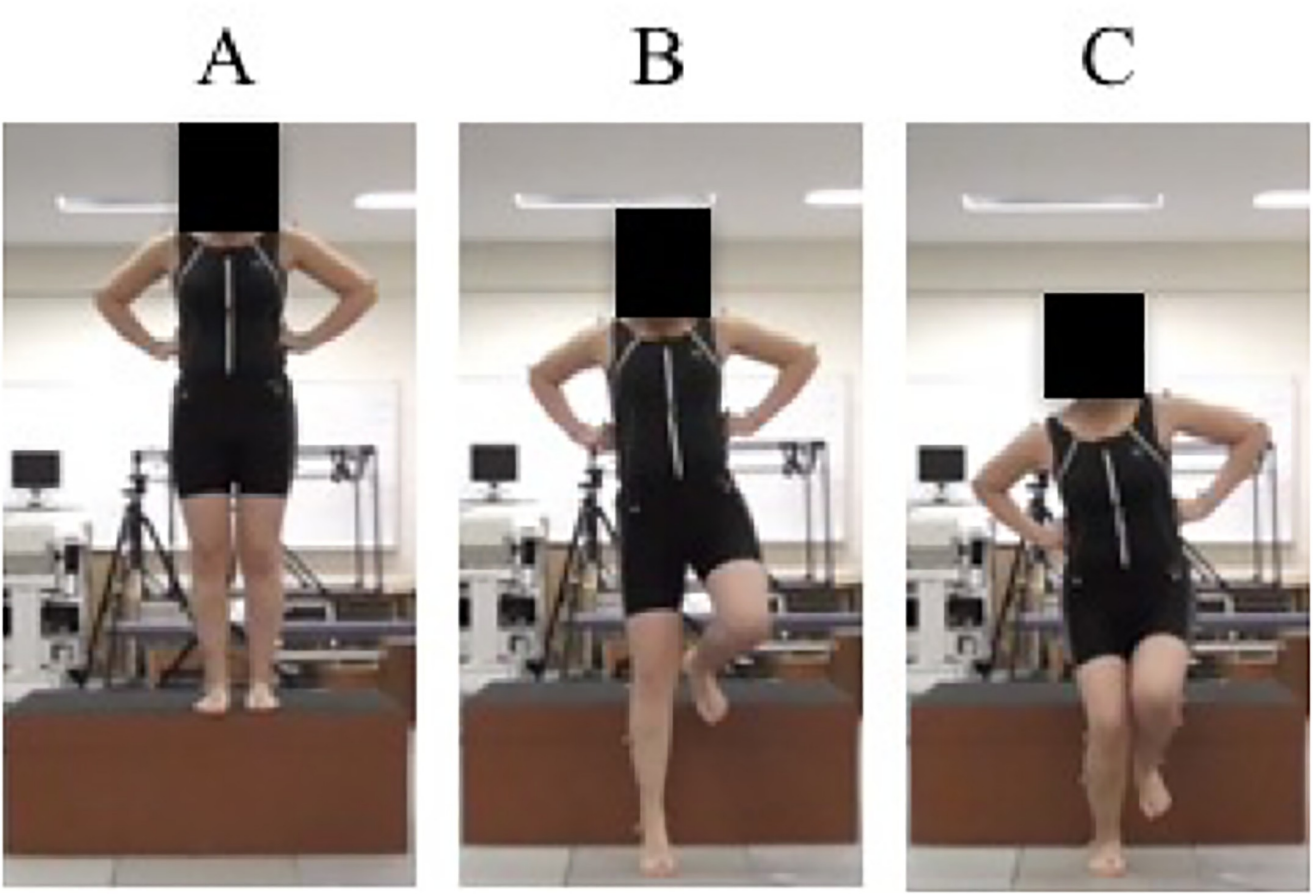
Each landing phase of single leg drop landing. (A) Single leg drop landing. Start position standing on a 40-cm box. (B) Initial contact on the dominant leg. (C)Subjects maintain the landing position.

The methods employed in evaluating SDL in this study were modified previous studies (Fig 1), [22,23]. All participants performed SDL on the dominant leg from a 40-cm box. SDL was used to categorise participants into a valgus group and a control group. Subsequently, they were asked to land with their dominant leg and to maintain their landing posture on the force plate for 2-seconds (s). All SDL tests were performed barefoot and with the hands of each subject placed on their lower rib cage. All participants performed three practice trials before measurements were taken. A failed trial was defined a case where participants were not able to maintain the landing posture for 2 s or landed on both legs. In such cases, they were asked to repeat the activity until three successful trials were completed.

### Star Excursion Balance Test

**Fig 2:**
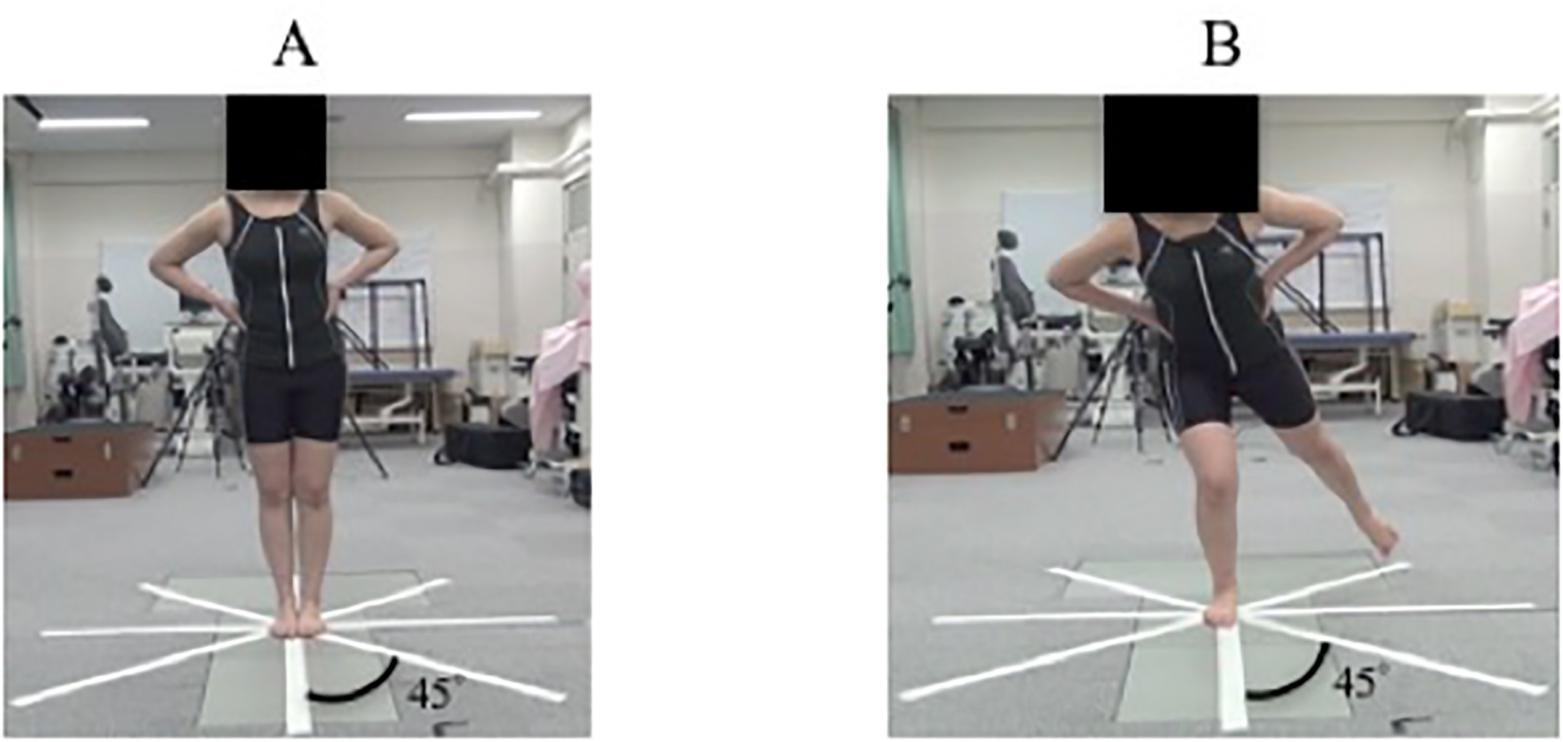
The Star Excursion Balance Test. (A) The Star Excursion Balance Test (SEBT). (B)Start position is standing. The SEBT was performed in single leg standing on the dominant leg at the centre of a grid pattern with lines drawn on the ground at 45 degrees to each other.

All participants performed the SEBT (Fig 2) [14] with the subject standing on their dominant leg at the centre of 8 grid lines drawn on the floor angled at 45 degrees to each other. The eight lines were labelled anterior (A), anterior medial (AM), medial (M), posterior medial (PM), posterior (P), posterior lateral (PL), lateral (L) and anterior lateral (AL). The subjects maximum reach and return back to the start position had to be accomplished in 3 s. All SEBTs were also performed barefoot, again with the hands of each subject placed on their lower rib cage. The verbal instructions during maximum reaching were to touch with their toes. All participants practiced their maximum reach in each direction three times before the tasks began. The SEBT defines three failure possibilities. First, where a part of the supporting leg planter separates from the floor surface. Secondly, where maximum reach and return back to start position cannot be accomplished in 3 s. Finally, a situation where the supporting leg or the trunk sways significantly during the trial. Three successfully completed tasks were required to complete the measurements of the SEBT.

### Data collection

The software VICON NEXSUS (Vicon MX system, VICON Motion System, Oxford, UK) was used for data analysis. After the knee angle in the frontal plane was calculated to categorise knee valgus or varus alignment during SDL, the three-dimensional angles of the trunk, hip and knee were calculated for each direction of the SEBT. The knee angle during SDL was defined as the difference in knee angle between that at the initial contact and at the maximum knee valgus or varus. Two force plates (MSA-6 Mini Amp, AMTI, MA, USA) were used to calculate the initial contact. The initial contact was defined as the point where 10 N of GRF was noted after landing. The mean knee valgus angle was calculated from 3 repeated trials and categorised the subject into either the valgus group or the control group based on a cut-off point of 5 degrees knee valgus angle, according to Olsen et al. [2]

The trunk, hip and knee angles during the SEBT were defined as the difference in each joint angle between that at the start position and that at maximum reach. The mean of each joint angle was calculated from three successful trials.

### Statistical analysis

All data were analysed using SPSS statistics version 23.0 for Mac OS (IBM). Independent t-tests were used to identify the difference in physical characteristics between the valgus group and the control group. Similarly, independent t-tests were used to identify the difference in the trunk, hip and knee angles during the SEBT between the two groups. The significance level was set at P <.05 for all analyses.

## Results

### Physical characteristics

There was no significant difference in the physical characteristics between the two groups. The physical characteristics and the knee angle in the frontal plane during SDL in the valgus group and the control group are shown in Table 1. The knee valgus angle during SDL was significantly greater in the valgus group (−8.3° ± 3.0°) than in the control group (4.6° ± 6.0°; P <.05).

### Knee valgus angle during the SEBT

The knee valgus or varus angles during the SEBT are shown in Table 2. The knee valgus angles in the valgus group were greater than those in the control group during reach directions: A (valgus group, −7.4° ± 8.5°; control group, 5.0° ± 5.5°), AM (valgus group, −7.7° ± 8.3°; control group, 3.0° ± 8.2°), M (valgus group, −10.9° ± 9.1°; control group, −0.2° ± 9.5°), PM (valgus group, −7.1° ± 9.9°; control group, 5.9° ± 7.0°) and AL (valgus group, −9.7° ± 8.1°; control group, 3.3° ± 7.2°; P < 0.05). The knee varus angles in the valgus group were lower than those in the control group during the reach directions of P (valgus group, 1.0° ± 10.0°; control group, 15.3° ± 8.2°), PL (valgus group, 11.7° ± 8.0°; control group, 23.8° ± 7.4°) and L (valgus group, 15.3° ± 8.7°; control group, 26.5° ± 7.0°; P < 0.05).

**Table 2:** The results of knee angle (mean ±SD, deg) in all reach directions during Star Excursion Balance Test.

Reach direction	Group	Knee angle ^a^					
		Sagittal plane		Frontal plane		Horizontal plane	
		Mean ± SD	P value	Mean ± SD	P value	Mean ± SD	P value
A	Valgus	49.1 ± 12.1	0.17	−7.4 ± 8.5	<0.001^c^	10.6 ± 5.3	0.44
	Control	55.0 ± 9.5		5.0 ± 5.5		12.3 ± 5.5	
AM	Valgus	54.0 ± 11.8	0.08	−7.7 ± 8.3	<0.001^c^	11.3 ± 8.2	0.39
	Control	61.3 ± 9.3		3.0 ± 8.2		13.8 ± 6.7	
M	Valgus	56.7 ± 13.1	0.04^b^	−10.9 ± 9.1	<0.001^c^	13.7 ± 7.3	0.43
	Control	66.5 ± 10.4		−0.2 ± 9.5		15.9 ± 6.8	
PM	Valgus	51.9 ± 12.8	0.34	−7.1 ± 9.9	<0.001^c^	12.4 ± 6.5	0.49
	Control	58.7 ± 13.1		5.9 ± 7.0		14.3 ± 7.2	
P	Valgus	45.5 ± 14.4	0.41	1.0 ± 10.0	<0.001^c^	7.3 ± 6.5	0.32
	Control	49.6 ± 11.6		15.3 ± 8.2		9.7 ± 5.8	
PL	Valgus	33.3 ± 11.3	0.10	11.7 ± 8.0	<0.001^c^	−2.5 ± 6.5	0.38
	Control	35.1 ± 10.4		23.8 ± 7.4		−0.3 ± 5.8	
L	Valgus	29.3 ± 11.0	0.25	15.3 ± 8.7	<0.001^c^	−8.6 ± 7.5	0.69
	Control	24.4 ± 10.3		26.5 ± 7.0		−9.7 ± 5.9	
AL	Valgus	39.7 ± 7.4	0.21	−9.7 ± 8.1	<0.001^c^	13.2 ± 5.5	0.38
	Control	44.7 ± 10.9		3.3 ± 7.2		11.4 ± 4.6	

^a^ The positive values are knee flexion (sagittal plane), varus (frontal plane) and internal rotation (horizontal plane), whereas the negative values are knee extension (sagittal plane), valgus (frontal plane) and external rotation (horizontal plane).

^b^ Statistically significant at p < 0.05.

^c^ Statistically significant at p < 0.001.

### Other joint angles during reach directions of A, AM, M, PM and AL

We also compared the angles of the trunk, hip and knee in the valgus group with those in the control group in the reach directions of A, AM, M, PM and AL during the SEBT (Tables 2–4). Trunk right rotation in the valgus group was lower than that in the control group during the reach direction of PM (valgus group, 2.1 ± 3.7°; control group, 5.7 ± 3.5°; P <.05) (Table 3). Hip internal rotation angle in the knee valgus group was lower than that in the control group during the reach direction of AM (valgus group, 2.7 ± 3.0°; control group, 5.7 ± 3.5°) and AL (valgus group, 5.6 ± 2.4°; control group, 8.4 ± 3.4°; P <.05) (Table 4). Furthermore, the knee flexion angle in the knee valgus group was lower than that in the control group during the reach direction of M (valgus group, 56.7 ± 13.1°; control group, 66.5 ± 10.4°; P <.05) (Table 2). In the reach direction of A, the knee valgus angle in the knee valgus group was greater than that in the control group, but there was no significant difference between the groups in other angles at the trunk, hip and knee joint.

**Table 3:** The results of trunk angle in all reach directions during Star Excursion Balance Test.

Reach direction	Group	Trunk angle (degree) ^a^					
		Sagittal plane		Frontal plane		Horizontal plane	
		Mean ± SD	P value	Mean ± SD	P value	Mean ± SD	P value
A	Valgus	8.2 ± 3.3	0.11	1.6 ± 2.4	0.89	0.4 ± 1.9	0.63
	Control	5.4 ± 4.8		1.8 ± 4.4		0.9 ± 2.9	
AM	Valgus	9.2 ± 2.9	0.47	−0.9 ± 2.9	0.82	2.7 ± 2.0	0.56
	Control	8.1 ± 4.5		−0.6 ± 4.2		3.3 ± 2.5	
M	Valgus	2.7 ± 3.4	0.09	−3.7 ± 2.4	0.47	3.9 ± 2.0	0.32
	Control	6.1 ± 5.4		−2.5 ± 6.5		4.8 ± 2.3	
PM	Valgus	−2.1 ± 5.2	0.22	−0.4 ± 3.9	0.83	2.1 ± 3.7	<0.01^c^
	Control	1.0 ± 6.7		0.0 ± 5.4		5.3 ± 2.0	
P	Valgus	−5.3 ± 4.6	0.049^b^	5.2 ± 3.1	0.05	0.8 ± 2.3	0.96
	Control	0.0 ± 9.1		8.4 ± 4.5		0.9 ± 2.8	
PL	Valgus	−2.5 ± 7.1	0.24	15.3 ± 3.8	0.82	1.4 ± 4.1	0.76
	Control	0.8 ± 6.8		14.8 ± 5.6		2.0 ± 4.9	
L	Valgus	−0.7 ± 5.1	0.55	17.0 ± 4.1	0.79	3.2 ± 3.9	0.15
	Control	0.7 ± 6.3		16.6 ± 4.7		5.4 ± 3.7	
AL	Valgus	4.8 ± 4.4	0.13	5.3 ± 2.9	0.54	−1.5 ± 3.3	0.15
	Control	1.7 ± 5.2		6.3 ± 5.1		−4.9 ± 6.6	

^a^ The positive values are trunk flexion (sagittal plane), side flexion (frontal plane) and rotation (horizontal plane) toward supporting leg, while the negative values are trunk extension (sagittal plane), side flexion (frontal plane) and rotation (horizontal plane) toward reaching leg.

^b^ Statistically significant at p < 0.05.

^c^ Statistically significant at p < 0.01.

**Table 4:** The results of hip angle in all reach directions during Star Excursion Balance Test.

Reach direction	Group	Hip angle (degree) ^a^					
		Sagittal plane		Frontal plane		Horizontal plane	
		Mean ± SD	P value	Mean ± SD	P value	Mean ± SD	P value
A	Valgus	10.4 ± 7.6	0.77	4.5 ± 6.6	0.70	2.3 ± 4.1	0.08
	Control	9.4 ± 8.0		5.2 ± 3.4		4.6 ± 2.6	
AM	Valgus	12.3 ± 7.4	0.83	1.5 ± 5.9	0.86	2.7 ± 3.0	0.031^b^
	Control	11.7 ± 6.3		1.1 ± 4.7		5.7 ± 3.5	
M	Valgus	28.4 ± 13.3	0.44	−4.6 ± 5.6	0.75	3.8 ± 4.4	0.45
	Control	31.6 ± 8.4		−3.9 ± 5.7		5.1 ± 4.3	
PM	Valgus	41.7 ± 13.4	0.34	−2.0 ± 7.5	0.56	6.0 ± 5.6	0.84
	Control	46.1 ± 10.5		−3.7 ± 7.0		5.6 ± 5.1	
P	Valgus	46.3 ± 15.4	0.20	9.1 ± 6.8	0.41	6.7 ± 5.5	0.89
	Control	52.9 ± 11.3		7.0 ± 6.3		6.4 ± 6.0	
PL	Valgus	40.2 ± 10.6	0.10	14.8 ± 4.6	0.65	5.3 ± 3.2	0.85
	Control	47.2 ± 10.1		13.8 ± 6.1		5.6 ± 7.2	
L	Valgus	35.2 ± 9.5	0.44	15.7 ± 4.0	0.30	3.4 ± 1.9	0.50
	Control	38.0 ± 8.9		13.7 ± 5.1		4.5 ± 6.2	
AL	Valgus	14.3 ± 9.4	0.58	11.6 ± 4.2	0.90	5.6 ± 2.4	0.035^b^
	Control	12.5 ± 7.1		11.8 ± 3.8		8.3 ± 3.4	

^a^ The positive values are hip flexion (sagittal plane), adduction (frontal plane) and internal rotation (horizontal plane), whereas the negative values are hip extension (sagittal plane), abduction (frontal plane) and external rotation (horizontal plane).

^b^ Statistically significant at p < 0.05

## Discussion

### Knee valgus angle during the SEBT

The knee valgus angle in the valgus group defined by SDL was significantly greater than that in control group during the SEBT in directions A, AM, M, PM, and AL. An increased knee valgus angle has been reported as a non-contact ACL injury risk factor by many previous studies [8–10,12–14, 24–26]. Evidence for this is provided by video motion analysis studies during non-contact ACL injury events [8, 10, 12, 13] and by three-dimensional analysis during cutting and landing movements [24–26]. Furthermore, Hewett et al. (2005) reported a cohort study in which increased knee valgus alignment during drop vertical jump put subjects at a higher risk for non-contact ACL injuries [14]. Interestingly, increased knee valgus alignment, which many previous studies report as posing a non-contact ACL injury risk, was similar to that seen in our study during the SEBT, in particular directions of A, AM, M, PM and AL. Therefore, increased knee valgus angle in directions A, AM, M, PM and AL during the SEBT may be considered as poor alignment which might be a factor contributing to non-contact ACL injury.

In addition to the increased knee valgus angle as a potential risk factor for non-contact ACL injury, our study also showed that subjects with increased knee valgus during SDL demonstrated a characteristic rotation alignment of the trunk and hip, together with knee flexion during the SEBT in directions AM, M, PM and AL. According to a previous study, alignment of the trunk and lower leg is important to evaluate when determining non-contact ACL injury risks [8]. Hence, we focused not only on knee valgus but also other body movements during the SEBT. In the following sections we discuss movement in 3 body regions during the SEBT.

### Trunk rotation angle during the SEBT in direction PM

Trunk rotation was significantly reduced in the valgus group during the SEBT when reaching in the direction of PM. Dempsy et al. reported an increased knee valgus with trunk rotation deficit to the supporting leg in subjects with various lower extremities conditions. Thus, indicating the potential that reduced trunk rotation might be a non-contact ACL injury risk factor [26]. The trunk rotation deficit to the supporting leg during the reach direction of PM was similar to the poor trunk control related to the pelvis suggested by Dempsy et al [25]. For this reason, we determined that this alignment causes a lack of knee joint stability due to a knee valgus alignment by changing the subject’s posture during the SEBT. Indeed, this alignment might be considered to be the opposite of what is needed to create the support for a stable lower extremity. Therefore, an increased knee valgus angle and decreased trunk rotation to the supporting leg in the valgus group when reaching in the PM direction is considered a characteristically poor alignment factor during an SEBT, potentially contributing to a non-contact ACL injury.

### Hip internal rotation angle in reach directions of AM and AL

Hip rotation during the SEBT in the directions AM and AL was significantly different between the two groups, with hip internal rotation decreased significantly in the valgus group. Consistent with this, Sigward et al. reported that increased hip internal rotation with an increased knee valgus might be a non-contact ACL injury risk factor based on three-dimensional motion analysis of cutting [24]. The reasons for these differences in the hip internal rotation angle in the valgus group were considered as follows. Cutting during running requires rapid deceleration and changing the centre of gravity which increases pelvis rotation on the thigh and thereby hip internal rotation. It appears that reaching in the AM and AL directions during the SEBT with an unstable knee joint helped enforce a posture with the pelvis in a neutral position to maintain the centre of gravity within a base of support; as a result, hip internal rotation is decreased. Previous studies of hip movement during cutting reported that the increasing pelvis rotation angle to the supporting leg increased the hip internal rotation angle [24], and that increasing hip internal rotation angle occurred concomitant with an increase in the knee valgus angle [25]. It is considered that the hip rotation angle depends upon the task because the kinematic characteristics vary according to the end rotation movement, and these differences were apparent in both previous studies and the present study findings. These findings suggest that an increased knee valgus angle and a decreased hip internal rotation angle identified in reach direction AM and AL during the SEBT might be a characteristically poor alignment potential identifying feature of non-contact ACL injury.

### Knee flexion angle in reach direction of M

Knee flexion angle during the SEBT while reaching in direction M was significantly different between the two groups, with hip internal rotation angle decreased significantly in the valgus group. Similar to the present studies finding’s, Koga et al. reported that an increased knee valgus angle with a decreased knee flexion angle at the time of non-contact ACL injury during sports might be a non-contact ACL injury risk factor, based on a photogrammetric Model-Based Image Matching method [13]. Because of the considerations mentioned above, an increased knee valgus angle and a decreased knee flexion angle identified in the M reach direction during the SEBT might be a potential identifying feature of non-contact ACL injury. During cutting and landing sports activity.

There are several limitations to our study. First, the participants in our study were categorised by knee valgus angle during SDL. Although this study used SDL which is a general dynamic alignment evaluation tool, in order to improve the usefulness of SEBT alignment evaluation, it is necessary to evaluate subjects with higher risk of non-contact ACL injury. Second, this study was a cross-sectional design. Future prospective studies should consider whether the SEBT can actually discriminate the non-contact ACL injury risk. We should take these factors into account when examining the usefulness of the evaluation of the alignment of the trunk and supporting leg during the SEBT.

## Conclusion

In this study, the subjects who had knee valgus greater than 5° during SDL showed a specific alignment of the trunk and supporting leg in the reach directions of A, AM, M, PM and AL during the SEBT. In addition to an increase in knee valgus in the reach directions of A, AM, M, PM and AL, our results showed that a decrease in trunk rotation to the supporting leg occurred during direction PM, with a decrease in hip internal rotation during directions AM and AL and a decrease in knee flexion during direction M. These findings may have value in identifying risk factors for non-contact ACL injury. Even though previous studies have reported that increased knee valgus angle combined with increased hip internal rotation angle are the main risk factor for non-contact ACL injury [24, 25], our results indicate that injury risk could be potentially picked up by evaluating the SEBT in reach directions AM and AL as these directions increased knee valgus angle combined with decreased hip internal rotation angle. The slow nature of the SEBT might provide easier evaluation of trunk and leg control than the relatively fast task of jump landing. Our results suggest that characteristic poor alignment of the trunk and the supporting leg during the SEBT in directions A, AM, M, PM and AL should be evaluated for each reach direction, and that these examinations might be useful in detecting ACL injury risk.
